# Systemic innovation for operationalising bioeconomy: A qualitative content analysis

**DOI:** 10.1016/j.heliyon.2024.e35914

**Published:** 2024-08-12

**Authors:** Laura Carraresi

**Affiliations:** Nofima AS, Department of Innovation, Consumer and Sensory Sciences, Osloveien 1, Ås, Norway

## Abstract

The purpose of the paper is to explore the attributes of systemic innovation that align with bioeconomy. The potential of systemic innovation, an overarching concept based on cooperation and integration along and across (emerging) value chains, to drive transitions towards bioeconomy has received limited attention. Therefore, this study aims to address this gap in knowledge. Through a systematic literature review followed by qualitative content analysis, we examine the drivers and obstacles to implementing a bio-based economy and explore which elements of systemic innovation could best facilitate these transitions. Findings suggest that systemic innovation facilitates the operationalisation of the bioeconomy by uniting stakeholders from diverse sectors and encouraging the acquisition of complementary knowledge, crucial factors in fostering innovation and driving disruptive changes. This enhances bioeconomy efficiency and competitiveness against fossil-based economies. However, systemic innovation is not a singular concept; rather, it encompasses various systemic characteristics, highlighting the need for concept harmonisation. The paper synthesises existing literature on systemic innovation in the bioeconomy, providing a framework for interdisciplinary research. By incorporating diverse systemic approaches into bioeconomic innovation, it bridges disciplinary divides and identifies key considerations for analysing sustainability transitions.

## 1 Introduction

### 1.1 Background

The journey towards sustainability across all sectors of the economy requires interventions at various levels to accomplish the desired global transformation. The United Nations Sustainable Development Goals [1] serve as a light guiding this systemic shift at the macro level. However, to attain these goals, actions must commence at the micro and meso levels. Thus, it is feasible to delineate a spectrum from ‘very weak’ to ‘strong’ sustainability [2,3], each marked by innovative solutions, ranging from those impacting individual companies to those progressively influencing value chains and reshaping systems.

Within this context, the bioeconomy is recognised as a potential remedy to global challenges [[4], [5], [6]]. Nonetheless, as argued by Vivien et al. (2019), there exist diverse interpretations of the bioeconomy: from a macro-level ecological perspective (Type I bioeconomy) striving for economic growth in harmony with biological evolution [7], to a knowledge-based bioeconomy (Type II bioeconomy) centred on a new paradigm rooted in biotechnology and innovation diffusion [8], and finally, to a biomass-based bioeconomy (Type III bioeconomy) aiming to substitute fossil resources with biological ones and linking both green and blue ecosystems to supply food, feed, bio-based products, energy, and services (European Commission, 2018 [9]). Each type of bioeconomy contributes to different sustainability paradigms ranging from ‘very weak’ to ‘strong’ sustainability [2].

This study concentrates on the Type III bioeconomy, which, as posited by Vivien et al. (2019) [10], can help address challenges of ‘weak sustainability’ by primarily engaging value chain actors (at the meso level) in devising sustainable biomass utilization solutions, albeit without directly confronting global-level challenges. Nonetheless, throughout the paper, we will retain the term ‘bioeconomy’, even though we specifically refer to Type III.

To operationalise bioeconomy, innovation^1^ is key [6,12], as it will allow value creation in the field through new products, processes or organisational practices, which are efficient alternative solutions to fossil-based products and have positive impacts on the environment and society at large.

In the scientific literature on environmental sciences and sustainability, continuous emergence of new concepts aims to define innovations that drive sustainability transitions. These concepts contribute differently to sustainability depending on their application scale [2]. For instance, sustainability-oriented innovations [13,14] and eco-innovations [[15], [16], [17]] operate at the micro level, focusing on technological and industrial solutions to enhance business sustainability while ensuring profitability. Co-innovation emphasises social learning and project monitoring, particularly in agricultural settings [[18], [19], [20]]. Supply chain innovation involves processes enabling firms to adapt to environmental uncertainty by modifying internal organisation and relationships among chain actors [[21], [22], [23], [24]].

At the meso level, innovation systems, whether national or regional, define networks of actors whose interactions facilitate knowledge flows and exploitation of complementary capabilities [[25], [26], [27]]. Innovation platforms catalyse multi-actor configurations via participatory processes [[28], [29], [30]], while innovation ecosystems involve chain actors and external stakeholders collaborating closely towards innovations [[31], [32], [33], [34], [35]] contributing to ‘very weak’ or ‘weak sustainability’, the positive environmental effects being the consequence of the technology developed, not the priority aim [2].

System innovations at the macro level endeavour to address global challenges and system changes [36], progressing towards ‘strong sustainability’ [2]. Dedicated innovation systems extend beyond technological systems, encompassing social and ethical considerations to foster system transformation [[37], [38], [39]].

### 1.2 Research gap and objectives

In discussions about sustainability and bioeconomy, researchers have often associated the term "systemic innovation" and/or the adjective “systemic” to the term "innovation system(s)" or other type of sustainability-oriented innovations, generating confusion and overlooking what "systemic innovation" actually is [13,39,40]. In particular, there tends to be a lack of exploration into the specific managerial attributes inherent in these terms and their role in facilitating transitions towards sustainability. The concept of ‘systemic innovation’ originates from the management literature and is defined as innovation that ‘requires significant readjustments to other parts of the system’ [41]. Taylor (2005) [42] argues that systemic innovation enhances existing products, but it demands adjustments to current processes and subsequent changes and adaptations to the operations of all affected companies. Systemic innovation enables synergistic value creation in tandem with related complementary innovations, extending beyond the boundaries of individual firms [[43], [44], [45], [46], [47]]. This phenomenon aligns well with bioeconomy transitions, where technological innovations are sought to recover scarce resources, reuse by-products, or implement recycling flows within the chain. Therefore, companies must embrace this systemic approach to collaborate, share information and resources, integrate and network with academics, stakeholders, and end-users to develop a comprehensive collection of multidisciplinary and inter-industry expertise [43,[48], [49], [50]].

Referring to various frameworks analysing innovations contributing to sustainability transitions, systemic innovation marks the initial and crucial phase of change, commencing with the focal company and gradually impacting other actors in the value chain and the wider system. Therefore, it operates at the micro-meso level, contributing to ‘weak sustainability’ [2], and serves as a catalyst for progress towards system transformation.

**Fig. 1:**
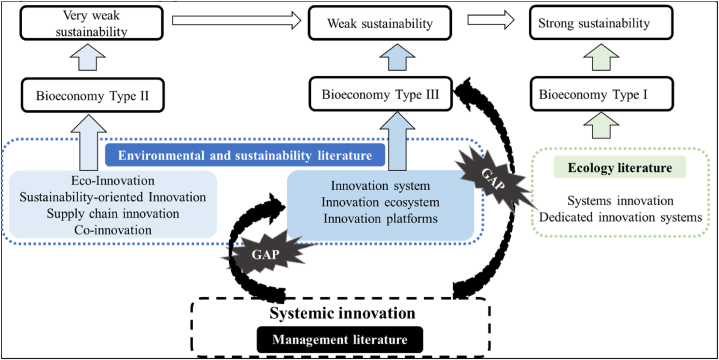
– Research gaps.

Source: Author's own elaboration based on [2,10,13,15,20,23,26,29,32,37].

To our knowledge, the concept of systemic innovation has not previously been applied to bioeconomy transitions, despite complementing several innovation types [12] at the micro and meso levels in the field. It appears that the concept of systemic innovation, originating from management literature, is inadvertently influencing the environmental and sustainability literature, despite being the precursor. Indeed, many aspects of systemic innovation are included in various types of innovations contributing to bioeconomy and sustainability, as mentioned earlier, without explicit recognition. Therefore, our aim is to clarify and enhance this literature by identifying the intrinsic characteristics of systemic innovation in order to understand if it can facilitate the operationalisation of the bioeconomy. Fig. 1 illustrates existing research gaps, and against this backdrop, the paper aims to address the following research question: *Which factors does systemic innovation influence to facilitate the operationalisation of the bioeconomy?*

The paper aims to achieve three main objectives: i) examining the concept of systemic innovation in scientific literature and emphasising its significant aspects, ii) delineating the challenges and drivers of bioeconomy implementation to derive transition requirements, and iii) investigating the correlation between systemic innovation and bioeconomy, identifying pivotal systemic innovation elements crucial for the bioeconomy.

To this end, we conducted a systematic literature review [21,51,52] and further engaged in qualitative content analysis (QCA) [53,54] to summarise existing literature, extract relevant elements, and analyse them descriptively. This approach adopts a 'miner' perspective [55], aiming to conceptually organise literature streams on systemic innovation and the bioeconomy based on their core components. Through this, we identify and summarise common characteristics that facilitate transitions.

The paper contributes in several ways. Firstly, it synthesises current literature on systemic innovation in the bioeconomy context, laying a foundation for interdisciplinary research. Secondly, it streamlines and simplifies the framework for systemic innovation approaches for bioeconomy, bridging disciplinary divides such as management, environmental, and innovation sciences. Lastly, it highlights key aspects for analysing bioeconomy transitions, preventing the proliferation of disparate concepts and definitions.

The remainder of the paper is organised as follows: the methodology of the systematic literature review and QCA is outlined in the next section; section 3 presents the findings; sections 4, 5 respectively discuss and conclude the study, with suggestions for future research also provided.

## 2 Methodology

### 2.1 Systematic literature review

We carried out a systematic literature review [56,57] in two steps: first, a sample of papers in line with the research question was selected; second, this sample was further analysed through a QCA (see section 2.2). The search syntax for the systematic literature review was derived from a standalone literature review conducted through Google Scholar, which served to depict a preliminary status quo of the term ‘systemic innovation’ in the bioeconomy context^2^ [58]. The following search string was derived:

TOPIC: (system* innovat*) OR TOPIC: (collaborat* innovat*) OR TOPIC: (innovat* system*) OR TOPIC: (network* innovat*) OR TOPIC: (chain innovat*) OR TOPIC: (system* approach) OR TOPIC: (open innovat*) OR TOPIC: (cooperat* innovat*) OR TOPIC: (integrat* innovat*) OR TOPIC: (cluster innovat*) OR TOPIC: (multisectoral innovat*) OR TOPIC: (co-innovation) OR TOPIC: (system* problem) OR TOPIC: (system* instrument*) AND (TOPIC: bioeconomy* OR TOPIC: bio-based OR TOPIC: bio-economy OR TOPIC: circular economy OR TOPIC: sustainable economy).

**Fig. 2:**
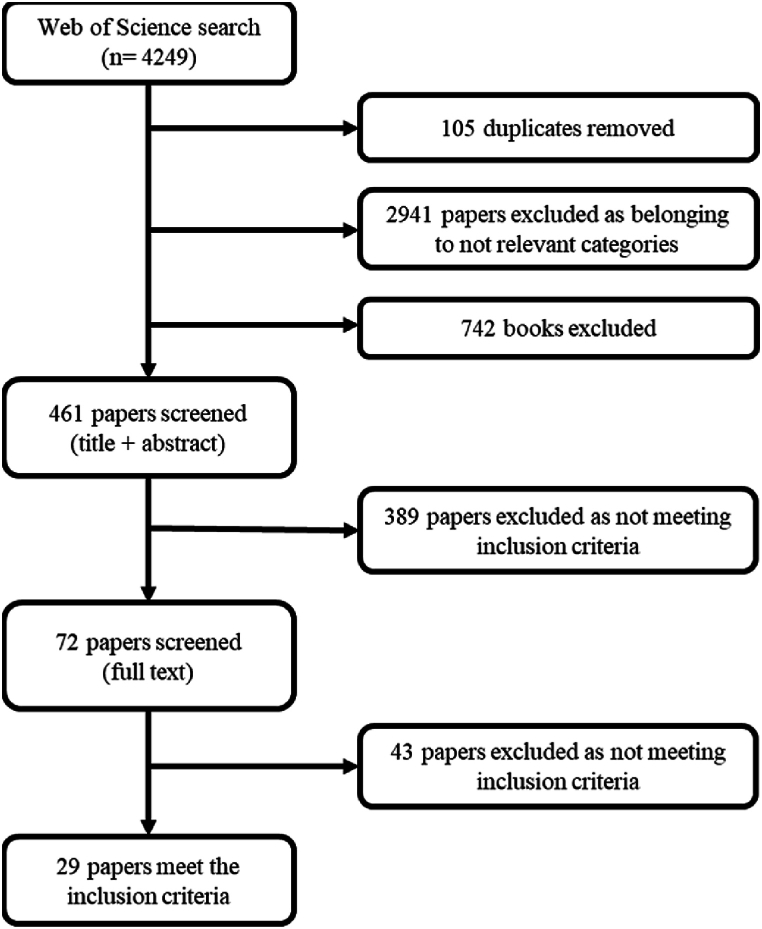
Selection procedure for the systematic literature review.

This search string was applied to source literature from the database Web of Science, which was chosen because it provides articles from journals with impact factor. The search was conducted in January 2019 by topic (title, abstract, keywords), with no time or language restrictions. Inclusion criteria are reported in Table 1, and the PRISMA statement was utilised for screening articles (Fig. 2) for their applicability to the paper's aim [59]. After screening, the final sample included 29 relevant articles.

**Table 1:** Inclusion criteria utilised for screening articles.

Type of document	Articles, Review articles, Proceedings papers are included.
Category/Subject	The article must be published in journals belonging to these categories: Environmental sciences, economics, business management, green sustainable science technology, environmental studies, engineering environmental, energy fuels, management, business, water resources, agricultural engineering, biotechnology, applied microbiology, multidisciplinary sciences, biodiversity conservation, engineering industrial, agriculture multidisciplinary, marine freshwater biology, agronomy and agricultural economics and policy.
Compliance with the definition of Systemic Innovation by Teece (1984) and Chesbrough and Teece (2008) [41] [43].	The innovation concept described or applied in the articles must comply with the definition from Teece (1984, p. 102) [41]: ‘[…] it requires significant readjustments to other parts of the system’, meaning that it is generated exclusively when accompanied by interrelated complementary innovations, and organisations are dependent on the other agents over whom they have no influence [43].
Compliance with the Bioeconomy Type III concept according to Vivien et al. (2019) [10]	The innovations described in the articles must refer to the conceptualisation of type III, as defined by Vivien et al. (2019, p. 192) [10]: ‘[…] is biomass-oriented, that oriented by the aim of ensuring transition to an economically viable use of biomass … the type III bioeconomy is united in its attempt to transform biomass from various sources. To deal with heterogeneous knowledge base, players use pilot and demonstration plant to determine possible bridging technologies and assess their maturity’.

### 2.2 Qualitative content analysis

**Fig. 3:**
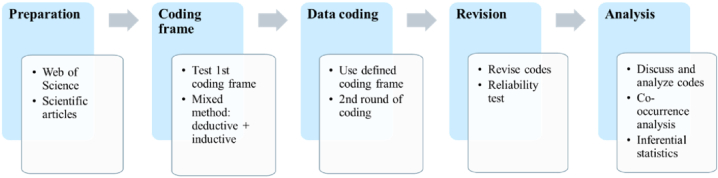
Qualitative content analysis: mixed approach.

Source: Author's own elaboration based on [62].

The final sample of papers underwent conceptual organisation and analysis through QCA, using the MAXQDA software with a single coder [60,61]. This method enables testing theoretical issues to gain deeper insights into the data and extract both manifest and latent content meanings [62]. Moreover, it allows condensing words into related categories, termed dimensions, to provide a concise description of a specific phenomenon. This method aligns well with the paper's objective, as it offers condensed yet essential knowledge on the subject with minimal loss of original data information [60]. MAXQDA software is widely used for QCA [63], offering advantages over manual analysis, including addressing potential weaknesses, such as the lack of transparency sometimes associated with QCA. Transparency can be enhanced by incorporating quantitative aspects into the analysis. MAXQDA also aids in pre-analysis, exploration, and result interpretation. The software's feature of progressively uploading documents during data collection facilitates immediate coding initiation. Additionally, its hierarchical association of codes with categories and the ability to quantify frequencies and cross-connections enable precise thematic analysis [64]. Supplementary tools, such as text systematisation, memo usage, colour coding for clarity, cross-referencing, and graph elaboration, further support this analysis, making MAXQDA a pertinent choice. The subsequent process, depicted in five steps (Fig. 3), occurred: 1) preparation, akin to the systematic literature review, involving paper unit selection; 2) coding frame development, employing an inductive and deductive approach [61,62] with a focus on data-driven code derivation based on the previous systemic literature review; here, a pilot testing phase is also carried out to cluster similar codes and create categories [65] 3) primary coding conducted in two rounds in different periods, utilising the established coding frame; 4) revision, incorporating reliability testing and Cohen's Kappa calculation (κ)^3^ to account for chance agreements [66,67]; and 5) analysis, evaluating category significance and code relationships to determine whether they occur together or near to each other and whether they are related.

Table 2 presents the coding frame utilised, while further information regarding code descriptions can be found in the Annexes. Parent codes and codes were employed, with subcategories introduced only when necessary to enhance conceptual clarity and establish connections with other analytical elements.

**Table 2:** – Coding frame and code system hierarchy.

Parent code	Code	Subcategory
Factors of systemic innovation	Level of relationship	Multi-stakeholder relationships/cross-industry nature/intra-industry collaboration/not specified collaboration
	Interdisciplinarity/complementarity/knowledge & technology management	
Trigger for innovation	Technology push/market pull/both	
Innovation phase	Conception/implementation/commercialisation	
Innovation type	Organizational/process & technology/business model/product or service	
Innovation context	Definitions/drivers/challenges	
Extent of change	Incremental/radical	
Innovation permeability	Open/closed/both	
Stakeholder participation	Organization/regional governments/policy makers/business or industry/research or academic institutions/user or consumer or communities/competitor/supplier/intermediaries/environment	
Bioeconomy context	Bioeconomy factors	Drivers/Challenges
	Circular economy definition/bioeconomy definition/approaches and initiatives/bioeconomy requirements	

From 29 academic papers, 1513 segments in total were coded throughout the entire process. Most of the codes relate to the main category, ‘Bioeconomy context’ (518 coded segments), representing 34.2 % of all codes. Meanwhile, the subcategory that contributed substantially was ‘Bioeconomy challenges’, totalling 219 segments (42 % of ‘Bioeconomy context’ codes). The second main category is ‘Factors of systemic innovation’, with 435 coded segments, whereas the subcategory ‘Multi-stakeholder relationships’ contributed the most, with 168 coded segments.

## 3 Results

### 3.1 Understanding the concept of systemic innovation in the bioeconomy domain

The systematic literature review revealed 23 distinct terms referring to systemic innovation within the bioeconomy domain, indicating a lack of consensus on its definition. Consequently, the definitions of systemic innovation diverge from Teece's original conception (1984) [41].

Key terms associated with systemic innovation in the bioeconomy domain identified through the literature review include.

• Innovation systems: These encompass networks of innovation, whether regional or national, involving various actors in the bioeconomy domain to develop new products, services, and business models. Such systems facilitate knowledge sharing and technology diffusion among stakeholders such as companies, policymakers, research institutions, and environmental groups. They dynamically interact towards a common purpose, leading to a lower market failure rate, enhanced societal acceptance, value addition, and regulatory compliance [68].
• Collaborative innovation: This umbrella term includes open innovation, network innovation, and systemic innovation itself. It denotes innovation arising from coordinated efforts among actors, involving changes along the value chain and the participation of multiple stakeholders. Innovations can span both within and beyond individual company boundaries [50,69,70].
• Sustainable innovation: Encompassing social innovation and eco-innovation, sustainable innovation aims to provide improved solutions to sustainability-related challenges, requiring collaboration and knowledge coordination at the development and design stages [71,72].

### 3.2 Category analysis

**Fig. 4:**
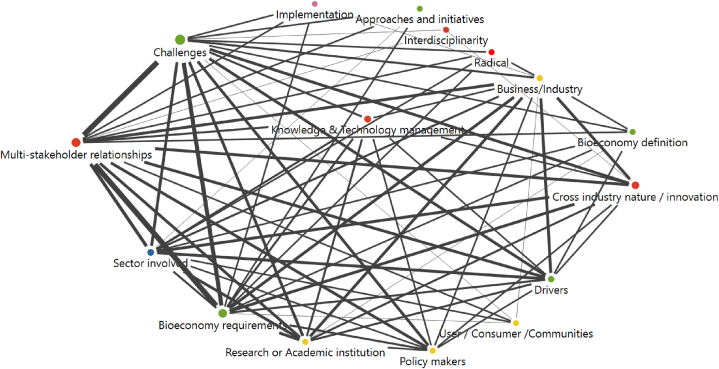
Code map relationships with frequencies and co-occurrences of coded categories.

A general overview of the codes' frequencies and the relations among them is shown in Fig. 4, including the co-occurrence of codes in the analysed literature. For better visualisation and readability, codes with less than 13 co-occurrences have been omitted from the graph, and different colours identify the codes according to their respective coding category, whereas the circle size indicates the code's frequency (see Annex 2 for detailed code frequencies). The connecting lines represent the co-occurrences between codes, whereas the thickness represents the frequency.

The sub-category with the highest code frequency and co-occurrence was ‘Multi-stakeholder relationships’, as the code interacts with both ‘bioeconomy requirements’ and ‘bioeconomy challenges’ and might explain the need for stakeholder collaboration to face bioeconomy requirements and challenges. This will be further analysed in the qualitative analysis phase.

#### 3.2.1 Factors of systemic innovation

The 'Factors of systemic innovation' category encompasses aspects that delineate the primary explanatory elements of systemic innovation. As per Teece's definition (1984) [41], innovations function systemically when they necessitate significant changes in practices across various actors along the value chain. Thus, relationships among actors and the extent of these interactions have been classified using the code 'level of relationship', resulting in four tiers of interaction: a) multi-stakeholder relationships; b) cross-industry collaboration; c) intra-industry collaboration; d) unspecified level of collaboration.

a. Relationships among actors of a different nature, such as institutions, organisations, firms, research institutions, universities, governmental bodies and international agents, are categorised as multi-stakeholder relationships. Compared to other levels, this category is frequently highlighted by scholars, as it facilitates knowledge sharing through mutual learning initiatives among actors, aiming to promote innovation. Moreover, addressing complex system challenges can be more effective through multi-stakeholder partnerships. Scholars suggest that firms should view themselves as coevolving actors within a broader system, where various interconnected agents develop innovations based on evolving societal needs. However, fostering reliable multi-stakeholder relationships requires appropriate cultural attitudes, collaborative skills, a systemic outlook, and openness to diverse disciplines. Thus, bioeconomy transitions can progress through co-evolutionary processes, involving institutional, technological, societal, ecological, and economic aspects interacting synergistically [73].
b. Cross-industry relationships frequently occurred in the literature analysed, wherein firms from different and hitherto unrelated sectors collaborated to develop and deploy innovations. The adoption of technologies at the intersection of various industries resulted in the formation of new value chains, necessitating cross-industrial partnerships for operation. Nevertheless, implementing such collaborations poses challenges due to the heterogeneity of involved firms, each with unique competencies that need integration.
c. Intra-industry relationships were rarely discussed in the analysed sample, involving partnerships within the same sector or among former collaborators. These existing relations enhance partnerships by fostering trust and facilitating knowledge sharing. As a result, firms aim not to expand collaborations indiscriminately but to cultivate efficient, profitable, cost-effective, and low-risk relationships. While intra-industry relationships typically demonstrate efficiency, they often lack the capacity to generate radical and disruptive innovations due to similarities in firm characteristics, competencies, and backgrounds [74].
d. Collaborations with non-specified actors were only captured by a few coded segments. Companies often seek to collaborate with various actors in the network rather than focusing solely on a select few stakeholders, making collaborations non-hierarchical [74].

The code 'interdisciplinarity' captures the need to draw upon knowledge from diverse fields to build competencies. Scholars acknowledge the importance of interdisciplinary approaches in innovation management processes to drive new technology development for bioeconomy [75]. Successful interdisciplinary approaches require an open organisational culture, particularly in fields such as biotechnology, life sciences, agronomy, ecology, food science, social science, nanotechnology, information and communication technologies, and engineering, which are deemed relevant to bioeconomy innovation processes.

The code 'complementarity' refers to the integration of diverse competencies, assets, and resources owned by different stakeholders to generate new technologies, knowledge, and innovations. It encompasses tangible and intangible resources, skills, know-how, infrastructures, technologies, research activities, market formations, production process enhancements, and financial support. Complementarity is evident in relationships where stakeholders are mutually dependent, possessing different resources or competencies essential for developing specific innovations (e.g., industrial symbiosis). Integrating novel technologies with existing industrial infrastructures enables synergies in energy and material flows and knowledge exchange. In cases where competencies are lacking, firms may establish strategic alliances to acquire them [76].

‘Knowledge and technology management’ encompasses fragments of the analysed documents detailing forms of knowledge and technology transfer, development, and administration. Given that innovative technologies from various industries drive the formation of new value chains and converging technologies facilitate horizontal integration across different sectors with distinct knowledge bases, effective knowledge and technology management is essential for establishing networks and synergies among stakeholders, scientists, policymakers, producers, and users.

#### 3.2.2 Trigger of innovation

The category ‘trigger of innovation’ examines whether innovations in the bioeconomy domain are technology-pushed or market-pulled, with findings indicating a predominance of the former. Research and development (R&D) play a crucial role, with biotechnology emerging as a pivotal enabling discipline. However, the literature also underscores the significance of responding to customer needs and developing new bio-based products in line with customer preferences. Transitioning towards bio-based economies necessitates the involvement of civil society, requiring consumers and end-users to participate in the ideation and development of new bioeconomy products [77].

#### 3.2.3 Innovation phase

Product innovations occurring in the bioeconomy field were categorised into three phases: 'conception' (early stage), 'implementation' (development stage), and 'launch'. During the early stages of innovation, knowledge gathering is paramount, involving multiple stakeholders in brainstorming and knowledge-sharing activities [50]. Subsequently, in the implementation phase, gathered knowledge and resources are utilised to develop and refine product innovations, preparing them for market entry. The finalisation of new products for launch and commercialisation may also involve subsequent process and/or organisational and business model innovations. Scholars suggest that challenges in the bioeconomy often arise during this phase, hindering further development and scaling activities. Regarding the launch phase, the literature generally concurs on the low technology readiness level of bioeconomy innovations, which gradually make their way to the market.

#### 3.2.4 Innovation type

The category ‘innovation type’ captures four different kinds of innovations observed in the bioeconomy domain: business model, process, product and organisational. Among them, only the first two were frequently mentioned in the literature as related to bioeconomy.

‘Business model innovation’ can derive from the adoption and scale-up of new bio-based technologies, requiring modifications to the value proposition, value creation or revenue model [78]. Being an innovation that involves internal procedures, it may also imply a lower cost incurred than other more cost-intensive innovation types.

‘Process innovation’ refers to the introduction of a new technology or method to improve process efficiency. In the bioeconomy domain, it encompasses clean technologies contributing to sustainability, such as recycling practices, by-product use and valorisation of waste streams. This type of innovation can also represent a precursor of new business models, as new technologies and processes often require a further reorganisation of key resources and activities or the involvement of new partners.

#### 3.2.5 Stakeholder participation and interaction

As outlined earlier, stakeholder interaction is a crucial determinant for bioeconomy transitions (refer to section 3.2.1). Therefore, a co-occurrence coding analysis was conducted to identify the most significant stakeholders and their interactions within the bioeconomy domain. The following actors were examined: industry stakeholders, research and academic institutions, policymakers, regional governments, public organisations, users and civil society, competitors, suppliers, intermediaries, and ecological representatives. From the analysis, three distinct clusters emerged based on the intensity of co-occurrence.

- High-intensity interaction: research and academic institutions, national or supra-national governmental authorities, policymakers, businesses and industrial actors.
- Middle-intensity interaction: civil society, consumers, users, environmental representatives, suppliers and public organisations.
- Low-intensity interaction: regional governments and intermediaries.

The analysis indicated that industry actors, research institutions, and policymakers are the most interactive stakeholders, engaging in both single and multi-level interactions. The latter are particularly vital for bioeconomy transitions, facilitated by collaborative efforts among academia, industry, and policymakers, forming what is known as the 'triple helix' [79]. Additionally, interactions extend to 'quadruple' [77]and 'quintuple helixes' [80], respectively, involving civil society and ecological representatives.

#### 3.2.6 Bioeconomy context

In our investigation of the bioeconomy context, considerable attention has been dedicated to identifying the drivers and challenges associated with transitions, examined at both macro and micro levels. At the macro level, notable similarities exist between the identified drivers and challenges, making it challenging for scholars to discern clear distinctions. This may be attributed to the fact that overarching challenges act as catalysts necessitating the adoption of a bioeconomy model. These encompass environmental and sustainability concerns, population growth, resource scarcity, fluctuations in fossil fuel costs, food and energy security, and the impacts of climate change.

On the micro level, drivers include customer and market demand and the availability of technology, whereas challenges encompass financial constraints, perceived risks, missing incentives, partner integration, switching costs, and user acceptance of new products and technology. Financial resources are crucial for investing in new technologies, pilot testing, and implementing new processes to launch and commercialise bio-based products. Considerable investments are necessary until these products prove sustainable and contribute to economies of scale to compete with conventional alternatives, posing challenges, especially for small enterprises [48,81]. Additionally, competitive fossil resource prices and high switching costs remain significant hurdles for successful bio-based innovation implementation, contributing to associated risks. Misaddressed or absent governmental incentives can exacerbate challenges related to land use purposes (food vs energy), land acquisition by multinationals, local environmental impacts, and social conflicts [82]. The integration of and partnerships among actors and stakeholders within and across diverse industries are also identified as crucial challenges facing research and innovation [83], potentially leading to business redesigns, increased transaction costs, and time investment, particularly due to the cross-industry nature of bioeconomy [84]. Furthermore, responding to market needs may present challenges concerning consumer acceptance of new technologies, varying across application fields and perceived benefits. Consumption patterns and preferences are continually evolving, necessitating firms to be adaptive and flexible.

The code ‘bioeconomy requirements’ captures elements contributing to bioeconomy transitions. Successfully implementing bioeconomic principles and business models requires transformative change at the macroeconomic level, involving not only companies but also institutions, policymakers, governments, and civil society. Scholars emphasize the pivotal role of innovation management in the bioeconomy, advocating for increased investments in radical and disruptive innovations aimed at exploiting the value of by-products. Furthermore, as radical innovations demand knowledge from diverse fields for successful development and implementation, mutual learning, collaboration, public–private partnerships, and cross-industry collaborations are deemed essential. Additionally, literature underscores the significance of a supportive policy and regulatory framework, introducing incentives to boost innovations and technologies reaching the market.

## 4 Discussion: the contribution of systemic innovation to operationalise bioeconomy

**Fig. 5:**
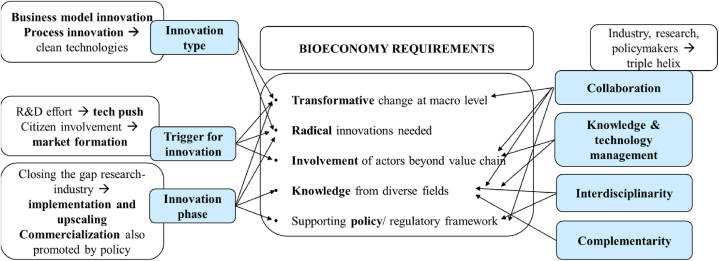
The contributions of systemic innovation to bioeconomy transitions.

Source: Author's own elaboration

The QCA facilitated the identification of key elements of systemic innovation essential for operationalising bioeconomy, thereby addressing the research question. Fig. 5 presents a synthesis of the findings from the literature review, illustrating the connections between the prerequisites for transitions in the bioeconomy and the elements defining systemic innovation, which could contribute to the transition if implemented.

Given the cross-industry nature of bioeconomy, a systemic action plan is imperative. This entails bridging knowledge disparities between research and industry and mobilising stakeholders across and beyond value chains to tackle complex sustainability challenges through co-evolutionary changes in technology, economy, culture, and organizational forms [73]. A participatory approach involving multiple stakeholders during the innovation process is vital, along with embracing diversity in knowledge sources and disciplines for developing new technologies in the bioeconomy sector, as affirmed by van Lancker et al. (2016) [50].

Three primary levels of collaboration are crucial here and could facilitate transitions. Collaboration among companies, government bodies, and academia, known as triple helix collaboration, enables the acquisition and generation of new knowledge, integration of diverse disciplines, and influence on R&D policies, thereby positively impacting innovation adoption and the introduction of new bio-based products in the market. It also aids in securing financial support and attracting new investors for bioeconomic initiatives.

Moreover, the co-occurrence analysis reveals intense interactions between civil society, governmental authorities, and businesses, indirectly fostering connections between triple helix actors and representatives concerned with environmental issues. Involving civil society in innovation activities introduces the 'quadruple helix' approach, contributing to a more significant societal transformation by facilitating changes in consumer behaviours, production patterns, technological advancements, norms, and values [77]. Interactions with users offer valuable insights to stakeholders about market demands. Therefore, consumers and society must be included in collaborations to ensure the long-term acceptance of novel goods and technologies. Informed consumers present both challenges and opportunities for firms to capture new market segments. However, differently from the framework on dedicated innovation systems (DIS) [37], systemic innovation per se does not aim to tackle societal issues, but rather to operationalise innovations through mutual dependence and collaboration between stakeholders, and interactions with consumers and users are aimed at achieving market success and profitability. We acknowledge that systemic innovation has a focus at micro and meso level and can only supports weak sustainability, whereas DIS have a wider horizon and aim at global sustainability transitions. Therefore, it could be possible that DIS originate from one or more systemic innovations, then spanning to consider social and ethical issues, but the opposite trend is rather difficult.

Aligned with Bauer et al. (2018) [74], collaboration plays a pivotal role in facilitating knowledge exchange and mutual learning, as well as in addressing the increasing complexity of technologies. Consequently, companies are encouraged to cultivate learning practices to enhance firms’ absorptive capacity, integrate external knowledge and expertise, and redesign business models to promote path-breaking, interdisciplinary, and open approaches [75]. Similarly, achieving optimal network performance may require the adjustment of roles by the involved parties. Conversely, excessive reliance on established paths and entrenched in prevailing business cultures can impede cross-industry collaboration and innovation [48,85,86].

Cross-industry relationships stem from the cascading utilization of resources, thereby contributing to transitions in the bioeconomy; hence, they are seen as prerequisites for establishing new value chains, catalysing industry convergence, and prompting structural adjustments where industry boundaries become blurred, reshaping the competitive landscape [48]. Multi-stakeholder and cross-industry collaborations are essential not only for fostering the conception and implementation of innovation but also for commercialisation. Particularly, suppliers and processors possess vital competencies and can bolster emerging technologies. However, the intense competition and complex market dynamics of bio-based products, often overshadowed by fossil-based alternatives, necessitate a systemic approach to commercialisation to facilitate market entry.

Another aspect linked to systemic innovation and characterised by cross-industry relationships is industrial symbiosis, which can also contribute to transitions in the bioeconomy. In this scenario, the systemic dimension is ingrained in the interdependence among distinct companies that exchange physical materials (such as water, energy, or by-products) essential for implementing specific technologies or innovations.

Our findings reveal that most of the elements defining systemic innovation and facilitating the operationalisation of the bioeconomy are also evident in numerous theoretical frameworks outlined in the background. Consequently, the concept of systemic innovation, originating from the domain of management literature, can be viewed as an antecedent to many of the innovation concepts emerging more recently within the sustainability literature. Bröring et al. (2020) [12] identified four primary types of innovation dominating transitions within bioeconomy. While they classify only new behaviours (innovation type IV) as systemic, our research demonstrates that systemic innovation characteristics are also applicable to new processes and products. Unlike the innovation system framework, systemic innovation encourages the practical implementation of innovations necessitating collaboration among multiple stakeholders, interdisciplinary approaches, and knowledge exchange. Thus, within the context of transitions in the bioeconomy, our review complements the innovation system framework by introducing additional elements crucial for innovation systems beyond mere interactions among network actors. Indeed, interdisciplinary collaboration, knowledge management and transfer, and adaptation of the innovation process during the conceptualisation and development phases of new bio-based products and processes are particularly pertinent, especially when they emerge at the interface of different industrial sectors, as seen in type III bioeconomy. Systemic innovations typically originate at the level of a focal company but subsequently require adjustments throughout the value chain and may give rise to emerging supply chains or innovation systems/ecosystems. Therefore, our analysis suggests that systemic innovations should be regarded as precursors to other innovation system frameworks applicable at the meso level. However, our study confirms that systemic innovation does not aspire to address global challenges at the macro level.

## 5 Conclusions

The present paper aimed to explore which elements of systemic innovation are pertinent to facilitating transitions from fossil-based to bio-based economies. This was achieved by conducting a QCA on a sample of 29 papers derived from a systematic literature review.

The key findings suggest that bioeconomy transitions pose challenges, requiring an overarching systemic change to overcome them. However, the direct usage of the term 'systemic innovation' is not prevalent in the bioeconomy literature; instead, various other terms have been identified, alluding to ‘systemic innovation’ with differing scales of application. Systemic innovation, therefore, serves as a precursor to the numerous streams of literature emerging in the environmental and sustainability realms.

Systemic innovation significantly contributes to successful bioeconomy transitions by fostering collaboration among stakeholders from different sectors in innovation development. For effective change, systemic innovation must operate at the multi-stakeholder or cross-industry level, catalysing disruptive shifts within the bioeconomy domain, thereby enhancing its efficiency and competitiveness against fossil-based products.

The study's limitations include potential interpretative biases due to predefined inclusion and exclusion criteria, as well as biases in the search strategy. Additionally, the reliance on the Web of Science database may have led to information loss, given its exclusion of certain scientific journals and grey literature. Furthermore, factors identified as both challenges and drivers of the bioeconomy (e.g., environmental topics related to climate change, sustainability issues, food and energy security, debates on political and regulatory support for bioeconomy transitions) may lead to misinterpretation. The time lag between data collection and publication also poses a limitation, potentially rendering some evidence outdated.

To address these limitations, future research should dig into intra-industry innovation alongside inter-sectoral innovation to better understand their respective impacts on sustainability transitions. Furthermore, assessing the perspectives of relevant stakeholders identified in this study could provide valuable insights into operationalising bioeconomy. Surveys and primary data analyses, such as Delphi studies and interviews, are recommended to gather empirical examples and expand the limited number of case studies in this domain. Lastly, an interdisciplinary approach between environmental and management sciences is proposed to leverage systemic innovation as a novel framework for addressing bioeconomy challenges, thereby averting the proliferation of new concepts and definitions.

## Funding

This research was funded by the Norwegian Foundation for Research Levy on Agricultural Products (Fondet for forskningsavgift på landbruksprodukter - FFL) through the project FoodForFuture (314318).

## Data availability statement

Data have not been deposited into a publicly available repository, but they will be made available on request, by contacting the author.

## CRediT authorship contribution statement

**Laura Carraresi:** Writing – review & editing, Writing – original draft, Visualization, Project administration, Methodology, Investigation, Formal analysis, Data curation, Conceptualization.

## Declaration of competing interest

The authors declare the following financial interests/personal relationships which may be considered as potential competing interests:Laura Carraresi reports statistical analysis was provided by Rheinische Friedrich Wilhelms University of Bonn Institute for Food and Resource Economics. If there are other authors, they declare that they have no known competing financial interests or personal relationships that could have appeared to influence the work reported in this paper.

## Figures and Tables

**Annex 1:** Code book

Parent Code	Description
1. Bioeconomy context	Definitions, approaches and factors that push and hinder bioeconomy development.
1.1 Bioeconomy factors	Factors that represent a challenge or pushes towards bioeconomy transitions and development.
1.1.1 Drivers	Factor that pushes bioeconomy development and transitions.
1.1.2 Challenges	Factor that hinders and should be solved to support bioeconomy development and transitions.
1.2 Circular Economy definition	Provided definitions of circular economy.
1.3 Bioeconomy definition	Provided definitions of bioeconomy.
1.4 Approaches and initiatives	Type of methods, strategies and stakeholder initiatives used to produce bioeconomic business models.
1.5 Bioeconomy requirements	Necessary conditions and factors required to implement bioeconomy processes and supporting transitions (fossil to bioeconomy).
2. Factors of Systemic Innovation	Factors that conform part of systemic innovation: innovations to be successful other parts of the value chain need to readjust, collaboration relationships at different scales, variety of disciplines (sciences), complementarity (of resources), knowledge and technology management.
2.1 Level of relationships	Collaboration relationships at different scales: multi-stakeholder, cross-industry and intra-industry collaborations.
2.1.1 Multi-stakeholder relationships	Collaborations occur at a multi-stakeholder level: cooperation between actors, institutions, organisations. e.g. business - academia, government - business.
2.1.2 Cross-industry relationship	Collaborations occur at a cross-industry level: firms of different and not previously related industries collaborate to create new products or value chains.
2.1.3 Intra-industry relationship	Collaborations occur at industry level: sense of collaboration between firms to achieve an innovation in one industry.
2.1.4 Not specified level of relationship	The level (scale) of the collaboration is not specified.
2.2 Interdisciplinarity	The combination of two or more sciences to develop an innovation.
2.3 Complementarity	Assets and resources that complement each other to create an innovation.
2.4 Knowledge & Technology management	Measures of knowledge and technology management, transfer and development during an innovation process.
3. Trigger for innovation	Factors that trigger innovation processes and developments. Technology or market factors?
3.1 Technology push	New technology and knowledge driven advances to foster innovation.
3.2 Market pull	Customer demands and preferences that drives new innovations.
3.3 Both	An innovation is triggered by technology or market factors.
4. Innovation phase	Innovation developments can be categorised in phases: Conception (early stage), Implementation (development stage), Commercialisation (market stage).
4.1 Conception	Innovation occurs in the early stages: knowledge creation, ideas, prototypes.
4.2 Implementation	Innovation occurs during the development and testing phases.
4.3 Commercialisation	Innovation occurs during the market launch and adoption of new product/service/business model.
5. Innovation type	The focus and type of innovation being pursued. What is being innovated exactly?
5.1 Organisational innovation	Organisational innovation covers the implementation of a new organisational method in the business practices, workplace organisation or external relations.
5.2 Process and Technology	Process innovation refer to the implementation of a new or significantly improved production or delivery method, while technology innovation englobes the development of novel technologies.
5.3 Business model	Change of an existing business model (value creation/delivery for customer) or the creation of a new business model that better satisfies the needs of the customer.
5.4 Product/Service innovation	The development and market introduction of a new, redesigned, or substantially improved good or service.
6. Innovation context	Encompasses innovation definitions, the necessary conditions to push innovation and factors that hinder or foster innovation.
6.1 Definitions	Definitions of types of innovations.
6.2 Drivers	Factors that foster innovations.
6.3 Challenges	Challenges, barriers, or constraints that hinder innovations to occur.
7. Extent of change	Degree of newness and level of disruptiveness of an innovation.
7.1 Incremental	Innovation that builds up on already existing developments representing a series of small improvements or upgrades made to existing products, services, processes or methods.
7.2 Radical	Innovation that destroys or supplants an existing business model, changes the market context and introduces a completely new technology/process/product.
8. Innovation permeability	Locus of an innovation: Inside our outside firms boundaries.
8.1 Open	Innovation occurs outside of an organisation with the collaboration of others.
8.2 Closed	Innovation happens inside of an organisation.
8.3 Both	Internal and external factors are critical for innovation developments.
9. Stakeholder participation	Actors that are involved in bioeconomy transitions and innovation processes.
9.1 Organisations	International, public and autonomous actors and institutions. e.g. NGO's, Associations involved in bioeconomy and innovation processes.
9.2 Regional governments	Political authorities limited to a local level: local municipalities, government involved in bioeconomy and innovation processes.
9.3 Policy makers	Political authority at a national or supra national level involved in bioeconomy and innovation processes.
9.4 Business/Industry	Firm or group of firms involved in bioeconomy and innovation processes.
9.5 Research or Academic institution	Universities or research institutions involved in bioeconomy and innovation processes.
9.6 User/Consumer/Communities	Encompasses civil society, users, and consumers of a product (or innovation), and local communities involved in bioeconomy and innovation processes.
9.7 Competitor	An organisation engaged in commercial or economic competition and who is involved in bioeconomy and innovation processes.
9.8 Supplier	Provider of raw material involved in bioeconomy and innovation processes.
9.9 Intermediaries	Actor that plays a role of supporter between different stakeholder relationships. e.g. brokers, consultants, banks, involved in bioeconomy and innovation processes.
9.10 Environment	Nature and environment considered as an actor involved in bioeconomy and innovation processes.
10. Sector involved	Industry involved in bioeconomy and innovation processes.

**Annex 2:**
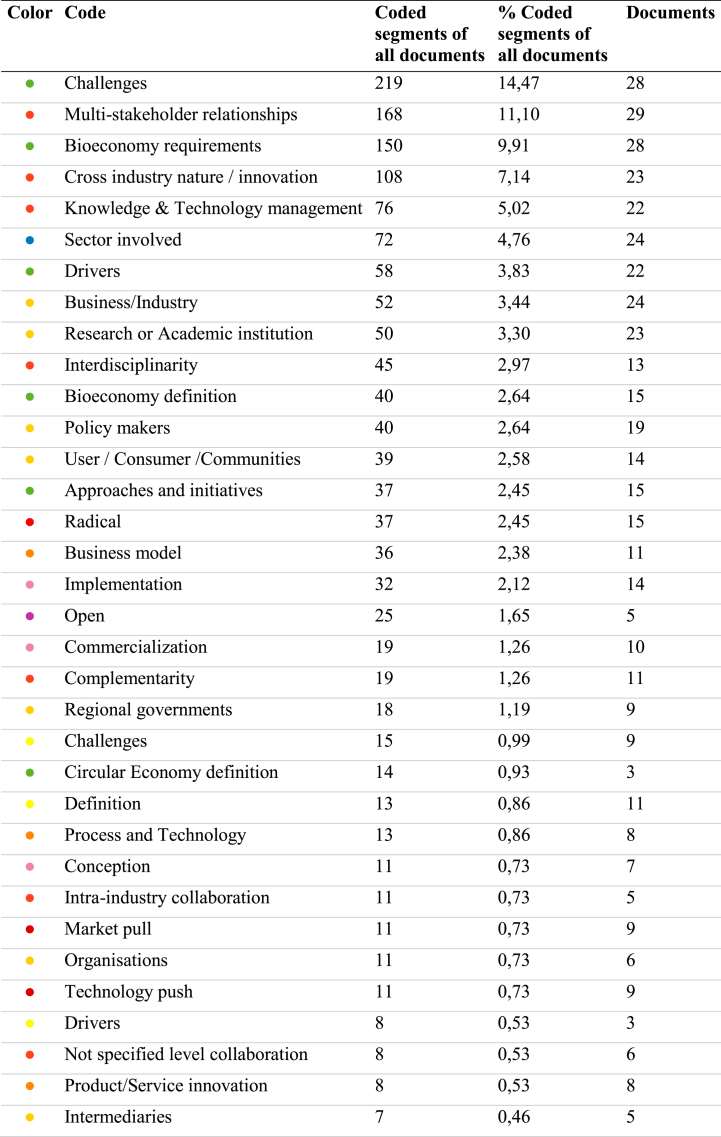

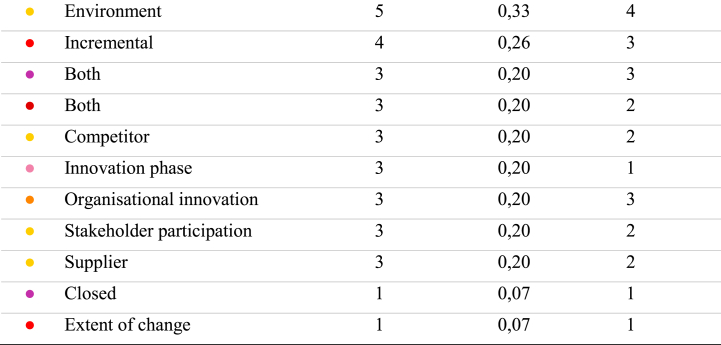
Overview of code frequencies

**Annex 3:** List of articles analysed

ID	Author	Title	Journal, Vol., pages	Year
#1	A. Giurca, P. Spath	A forest-based bioeconomy for Germany? Strengths, weaknesses and policy options for lignocellulosic biorefineries	Journal of Cleaner Production, 153, 51–62.	2017
#2	A. Tani	A Strategic Niche Management approach for shaping bio-based economy in Europe	Open Agriculture, 3 (1), 98–109.	2018
#3	A. Tsvetkova, M. Hellström, M. Gustafsson, J. Sjoblom	Replication of industrial ecosystems: the case of a sustainable biogas-for-traffic solution	Journal of Cleaner Production, 98, 123–132.	2015
#4	A.A.E. Pigford, G.M. Hickey, L. Klerkx	Beyond agricultural innovation systems? Exploring an agricultural innovation ecosystems approach for niche design and development in sustainability transitions	Agricultural systems, 164, 116–121.	2018
#5	B. Aquilani, C. Silvestri, G. Ioppolo, A. Ruggieri	The challenging transitions to bio-economies: Towards a new framework integrating corporate sustainability and value co-creation	Journal of Cleaner Production, 172, 4001–4009.	2018
#6	B. Cavicchi, S. Palmieri, M. Odaldi	The Influence of Local Governance: Effects on the Sustainability of Bioenergy Innovation	Sustainability, 9 (3), 406.	2017
#7	B. Golembiewski, N. Sick, S. Bröring	The emerging research landscape on bioeconomy: What has been done so far and what is essential from a technology and innovation management perspective?	Innovative Food Science & Emerging Technologies, 29, 308–317.	2015
#8	B. Baldassarre, M. Schepers, N. Bocken., E. Cuppen., G. Korevaar, G. Calabretta	Industrial Symbiosis: towards a design process for eco-industrial clusters by integrating Circular Economy and Industrial Ecology perspectives	Journal of cleaner production, 216, 446–460.	2019
#9	C. Ingrao, J. Bacenetti, A. Bezama, V. Blok, P. Goglio, E.G. Koukios, M. Lindner, T. Nemecek, V. Siracusa, A. Zabaniotou, D.Huisingh,	The Potential Roles of Bio-Economy in the Transition to Equitable, Sustainable, Post Fossil-Carbon Societies: Findings from this virtual special issue	Journal of Cleaner Production, 204, 471–488.	2018
#10	C. Scheel	Beyond Sustainability. Transforming industrial zero-valued residues into increasing economic returns	Journal of cleaner production, 131, 376–386.	2016
#11	D.Wield, R. Hanlin, J. Mittra, J. Smith	Twenty-first century bioeconomy: Global challenges of biological knowledge for health and agriculture	Science and Public Policy, 40 (1), 17–24.	2013
#12	D. Loorbach, J.C. van Bakel, G. Whiteman, J. Rotmans	Business Strategies for Transitions Towards Sustainable Systems	Business strategy and the environment, 19 (2), 133–146.	2010
#13	E. Hodgson, M.E. Ruiz-Molina, D. Marazza, E. Pogrebnyakova, C. Burns, A. Higson, M. Rehberger, M. Hiete, M. Gyalai-Korpos, L. Di Lucia, Y. Noël, J. Woods, J. Gallagher	Horizon scanning the European bio-based economy: a novel approach to the identifi cation of barriers and key policy interventions from stakeholders in multiple sectors and regions	Biofuels, Bioproducts and Biorefining, 10 (5), 508–522.	2016
#14	F. Bauer, T. Hansen, H. Hellsmark	Innovation in the bioeconomy – dynamics of biorefinery innovation networks	Technology Analysis & Strategic Management, 30 (8), 935–947.	2018
#15	H. Hellsmark, J. Mossberg, P. Söderholm, J. Frishammar	Innovation System Strengths and Weaknesses in Progressing Sustainable Technology: The Case of Swedish Biorefinery Development	Journal of Cleaner Production, 131, 702–715.	2016
#16	I. Grundel, M. Dahlström	A Quadruple and Quintuple Helix Approach to Regional Innovation Systems in the Transformation to a Forestry-Based Bioeconomy	Journal of the Knowledge Economy, 7, 963–983.	2016
#17	J. Mossberg, P. Söderholm, H. Hellsmark, S. Nordqvist	Crossing the biorefinery valley of death? A role-based typology for understanding actor networks' ability to overcome barriers in sustainability transitions	Environmental innovation and societal transitions, 27, 83–101.	2018
#18	J. Van Lancker, E. Wauters, G. Van Huylenbroeck	Managing innovation in the bioeconomy: An open innovation perspective	Biomass and Bioenergy, 90, 60–69.	2016
#19	L. Carraresi, S. Berg, S. Bröring	Emerging value chains within the bioeconomy: Structural changes in the case of phosphate recovery	Journal of Cleaner Production, 183, 87–101.	2018
#20	M. Boehlje, S. Bröring	The Increasing Multifunctionality of Agricultural Raw Materials: Three Dilemmas for Innovation and Adoption	International Food and Agribusiness Management Review, 14 (1030-2016-82773), 1–16.	2011
#21	N.R. Jordan, K. Dorn, B. Runck, P. Ewing, A. Williams, K.A. Anderson, L. Felice, K. Haralson, J. Goplen, K. Altendorf, A. Fernandez, W. Phippen, J. Sedbrook, M. Marks, K. Wolf, D. Wyse, G. Johnson	Sustainable commercialisation of new crops for the agricultural bioeconomy	Elementa, 4, 000081.	2016
#22	O. Segura-Bonilla	Competitiveness, systems of innovation and the learning economy: the forest sector in Costa Rica	Forest Policy and Economics, 5 (4), 373–384.	2003
#23	P. Theinsathid, A. Chandrachai, S. Keeratipibul	Managing Bioplastics Business Innovation in Start Up Phase	Journal of technology management & innovation, 4 (1), 82–93.	2009
#24	S. Silveira, F.X. Johnson	Navigating the transition to sustainable bioenergy in Sweden and Brazil: Lessons learned in a European and International context	Energy Research & Social Science, 13, 180–193.	2016
#25	S. Leipold, A. Petit-Boix	The circular economy and the bio-based sector - Perspectives of European and German stakeholders	Journal of cleaner production, 201, 1125–1137.	2018
#26	S. Wydra	Value Chains for Industrial Biotechnology in the Bioeconomy-Innovation System Analysis	Sustainability, 11 (8), 2435.	2019
#27	T. Marsden, F. Farioli	Natural powers: from the bio-economy to the eco-economy and sustainable place-making	Sustainability Science, 10, 331–344.	2015
#28	T.T. Sousa-Zomer, P.A. Cauchick-Miguel	Exploring business model innovation for sustainability: an investigation of two product-service systems	Total Quality Management & Business Excellence, 30 (5–6), 594–612.	2019
#29	V. Kargytė, R. Värnik, V. Aleknevičienė	Assessment of Bioeconomy Development Potential from the Perspective of Innovation Economics in European Regions	Management Theory and Studies for Rural Business and Infrastructure Development, 40 (4), 492–513.	2018

## Footnotes

1 Innovation is defined by the Oslo Manual (2005) as a new or improved product, process, marketing or organisational method. A product is considered an innovation when it becomes available on the market; meanwhile, the other innovation types must be implemented and operationalised in the firm [11].

2 The main focus is on bioeconomy development. Nevertheless, papers related to the circular economy domain have been also included in the systematic literature review to obtain a broader sample of works and to prevent any significant information from being overlooked.

3 Cohen's Kappa can range from −1 to +1, where 0 represents the amount of agreement that can be expected from random chance and 1 represents perfect agreement. Values equal to or lower than 0 represent no agreement, 0.01–0.20 no or minor agreement, 0.21–0.40 low agreement, 0.41–0.60 moderate agreement, 0.61–0.80 strong agreement and 0.81–1.00 as practically perfect agreement. Nevertheless, many scholars suggest a minimum of 0.80 agreement to be acceptable. Because the reliability assessment is intra-coder, meaning the coding comparison was performed across different points in time and by only one coder, a minimum of 0.80 agreement is expected. To calculate Cohen's Kappa, the following formula was used [66]: $\kappa =\frac{\mathrm{Pr}\left(a\right)-\mathrm{Pr}\left(e\right)}{1-\mathrm{Pr}\left(e\right)}$ where the actual observed agreements are represented by Pr(a), while Pr(e) refers to chance agreements.
